# Insulin-like Growth Factor 1 Ameliorates Intestinal Barrier Dysfunction in MASLD via IGF-1R/PI3K/AKT Signaling

**DOI:** 10.3390/nu18111667

**Published:** 2026-05-22

**Authors:** Wenshuo Zhao, Jishuang San, Fan Jiang, Yue Zhu, Gaofeng Wu, Jiancheng Yang, Weiwei Li

**Affiliations:** Liaoning Provincial Key Laboratory of Zoonosis, College of Animal Science & Veterinary Medicine, Shenyang Agricultural University, Shenyang 110866, China; z13942923144@163.com (W.Z.);

**Keywords:** IGF-1, intestinal mechanical barrier, IGF-1R/PI3K/AKT, metabolic dysfunction-associated steatotic liver disease, rat

## Abstract

**Background**: Metabolic dysfunction-associated steatotic liver disease (MASLD) represents a globally prevalent hepatic disorder, characterized by hepatic lipid accumulation and extrahepatic complications, notably intestinal barrier injury, which further exacerbates MASLD progression. The “gut–liver axis” has been identified as a critical contributor to MASLD development, with insulin-like growth factor 1 (IGF-1) serving as a pivotal coupling factor of this axis. However, the specific role and molecular mechanism by which IGF-1 modulates intestinal barrier function in the context of MASLD remains unclear. **Methods**: This study analyzed the correlations between the GH/IGF-1 axis and intestinal barrier function in MASLD rats, and explored the effects of IGF-1 intervention both in vivo and in vitro. **Results**: Our results showed that MASLD rats exhibited intestinal barrier impairment, characterized by elevated serum Diamine oxidase (DAO) and D-Lactate (D-LAC) levels, villus damage, and downregulation of tight junction proteins and Mucin (MUC2). These changes were accompanied by suppression of the GH/IGF-1 axis. Correlation analysis uncovered a negative association between IGF-1 levels and markers of barrier dysfunction. IGF-1 intervention effectively repaired the intestinal barrier structure of MASLD rats and significantly upregulated the expressions of IGF-1R, PI3K, and AKT. In vitro, IGF-1 treatment improved transepithelial electrical resistance (TEER), enhanced barrier-related gene expression, promoted cell proliferation, and inhibited apoptosis. **Conclusions**: These findings suggested that GH/IGF-1 axis suppression, intestinal barrier dysfunction, and IGF-1R/PI3K/AKT signaling were interconnected within the gut–liver axis in MASLD. IGF-1 may contribute to barrier regulation through associated signaling changes, highlighting the GH/IGF-1 axis as a potential complementary target.

## 1. Introduction

Metabolic dysfunction-associated steatotic liver disease (MASLD) has evolved into a global epidemic, posing a severe threat to human health. Meta-analysis data showed that the global prevalence of MASLD has risen from 25.3% between 1990–2006 and 38.0% in the 2016–2019 period [1], affecting approximately one-quarter of the global adult population and emerging as the most prevalent chronic liver disease in industrialized countries. More importantly, with the increasing incidence of obesity and metabolic syndrome, the prevalence of MASLD is expected to continue rising. It is estimated that over 50% of obese individuals and approximately 70% of T2DM patients are susceptible to MASLD development [2], indicating a strong bidirectional association between MASLD and these metabolic disorders. Although intrahepatic lipid accumulation is the hallmark of MASLD, the disease is essentially a multisystem metabolic disorder that not only causes hepatic injury but also increases the risk of extrahepatic organ damage and dysfunction, including intestinal barrier impairment, cardiovascular diseases, and the progression of existing metabolic complications [3].

Accumulating evidence has demonstrated that the “gut–liver axis” is a key pathogenic driver of MASLD progression, among which intestinal barrier dysfunction, as a core link in gut–liver axis dysregulation, is crucial to linking intestinal homeostasis imbalance, inflammatory responses, and metabolic dysfunction imbalance to metabolic dysfunction and hepatic inflammation [4]. The intestinal mechanical barrier, which consists of the mucus layer, epithelial tight junctions, and intact mucosal architecture, constitutes the primary line of defense against luminal antigens, toxins, and pathogens [5]. Notably, clinical studies have shown that compared with healthy controls, MASLD patients exhibit a 1.6-fold increase in serum diamine oxidase (DAO) and a 1.7-fold increase in D-lactate (D-LAC) [6]. Histopathological analysis of colonic biopsies further revealed a 40–60% reduction in the expression of ZO-1 and Occludin, accompanied by disruption of epithelial continuity [7]. This barrier damage promotes the translocation of bacterial endotoxins (such as LPS) and microbial metabolites (such as TMAO) into the systemic circulation, thereby activating TLR4/NF-κB signaling in the liver, triggering hepatic inflammation, and exacerbating steatosis; consistent with this mechanism, plasma LPS concentrations are significantly elevated in NASH patients relative to healthy individuals and positively correlated with hepatic lobular inflammation scores [8]. Therefore, restoring intestinal barrier function represents a pivotal interventional strategy to ameliorate the MASLD progression.

Insulin-like growth factor 1 (IGF-1), a central regulator of growth and metabolism, has recently been implicated in the maintenance of intestinal barrier function. As a pleiotropic polypeptide hormone primarily synthesized in the liver under the regulation of growth hormone [9], IGF-1 plays crucial roles in cell proliferation, differentiation, apoptosis and metabolic homeostasis [10]. Current research has substantiated that dysregulation of the GH/IGF-1 axis is closely associated with various metabolic disorders, including MASLD. Specifically, IGF-1 levels in the circulation and liver tissue are significantly reduced in MASLD patients, and although the reduction is correlated with disease severity [11], the specific mechanism through which IGF-1 protects the intestinal mechanical barrier in the context of MASLD remains poorly characterized. For instance, serum IGF-1 levels decreased by approximately 20% in patients with mild MASLD, whereas the reduction could reach 40–50% in those with severe MASLD compared with healthy individuals [12]. Similarly, in rodent models, High Fat Diet (HFD)-induced MASLD rats exhibit a downregulation of hepatic IGF-1 mRNA by approximately 45% and a reduction in serum IGF-1 by approximately 38% [13]. In addition, emerging evidence also indicates that IGF-1 exerts protective effects on multiple organ systems, including the intestine. For instance, in vitro studies have shown that pretreatment with 100 ng/mL IGF-1 can reverse LPS-induced barrier dysfunction, increasing transepithelial electrical resistance (TEER) by 2.1-fold and reducing FITC-dextran permeability by 40% [14]. In vivo experiments, administration of 22 μg/L IGF-1 to rats significantly upregulated the expression of MUC2 mRNA (a marker of intestinal chemical barrier, primarily secreted by goblet cells), which is likely attributed to IGF-1-mediated acceleration of goblet cell renewal, thereby ameliorating intestinal chemical barrier damage [15]. These protective effects have been reported to be associated with the activation of IGF-1R and downstream PI3K/AKT signaling pathways, which are known to regulate epithelial integrity, cell proliferation, and anti-apoptotic responses [16]. Notably, most prior investigations have primarily centered on the general protective role of IGF-1 on overall intestinal barrier integrity, whereas the specific role and underlying molecular mechanisms by which IGF-1 mitigates intestinal mechanical barrier injury in the context of MASLD conditions remain poorly understood.

Based on this, the present study was designed to investigate the interplay among GH/IGF-1 axis suppression, intestinal mechanical barrier dysfunction, and IGF-1R/PI3K/AKT signaling alterations within the framework of the gut–liver axis in MASLD. To achieve this, we established HFD-fed MASLD rat models (in vivo) and LPS-treated Caco-2 cell models (in vitro). Rather than focusing solely on the protective effect of IGF-1, this study aims to systematically characterize the association and potential relationship among GH/IGF-1 axis inhibition, barrier impairment, and downstream signaling changes under MASLD conditions. In addition, we explored whether exogenous IGF-1 intervention is associated with remodeling of these pathological alterations and partial restoration of intestinal barrier homeostasis. Overall, this study seeks to provide an integrated perspective on the GH/IGF-1 axis within the gut–liver axis context, rather than simply confirming a single signaling pathway, thereby offering a more comprehensive understanding of its potential role in MASLD progression.

## 2. Materials and Methods

### 2.1. Animals, Cells and Reagents

All animal experiments involved in this study were approved by the Animal Care and Use Committee of Shenyang Agricultural University (No. 2023090601). 30 male SPF-grade SD rats (6–8 weeks, 180–200 g) were purchased from Beijing Vital River Laboratory Animal Technology Co., Ltd. (Beijing, China). The human colorectal adenocarcinoma epithelial cell line (Caco-2) was sourced from Procell Life Science & Technology Co., Ltd. (Wuhan, China). IGF-1 was purchased from Novoprotein Scientific Inc. (Suzhou, China). LPS was acquired from Solarbio Technology Co., Ltd. (Beijing, China).

### 2.2. Design of Animal Experiments

**Figure 1:**
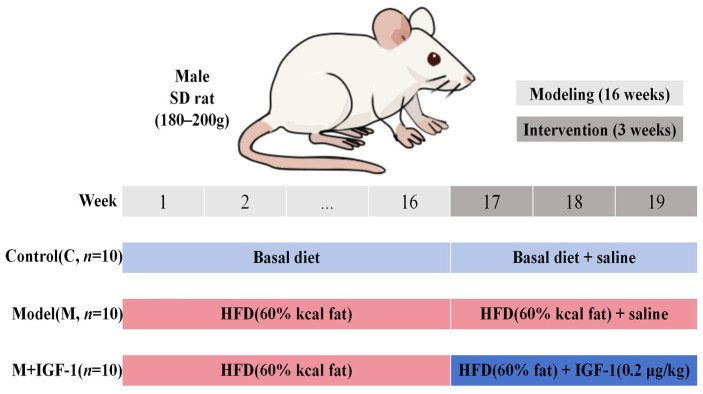
Animal experiment design flow. Initially, 10 rats were allocated to each group. Following rigorous quality control and pre-defined exclusion criteria assessment, 6 biological replicates (individual rats) per group were finally included in the statistical analysis.

The MASLD rat model was induced using the approach reported by Emamat et al. [9]. Thirty male SD rats were selected and housed under controlled conditions (25 °C, 50% relative humidity, 12 h light/dark cycle) with free access to water and food. After 1 week of adaptive feeding, the rats were randomly divided into 3 groups: the control group (C group, *n* = 10, basal diet + saline), the model group (M group, *n* = 10, HFD containing 60% kcal fat + saline), and IGF-1 intervention group (M + IGF-1 group, *n* = 10, HFD + daily subcutaneous injection of IGF-1 at 0.2 μg/kg body weight). Body weight was measured weekly during the 16-week modeling period, and this HFD protocol has been confirmed to induce typical MASLD pathological features [17,18]. When the experiment was finished, the rats were anesthetized and sacrificed via cervical dislocation. The serum, liver and ileum tissues were collected immediately for weighing and organ index calculation (organ weight/body weight × 100%). Sections of liver and ileum tissues were preserved in 4% paraformaldehyde for subsequent detection. Pre-defined exclusion criteria and quality control standards were established prior to statistical analysis. Animals were excluded from the final analysis if they met any of the following conditions: accidental death unrelated to experimental procedures, incomplete sample collection, or tissue samples that failed to meet quality control requirements. Following this rigorous assessment, 6 biological replicates (individual rats) per group were finally included in the statistical analysis. The composition of the 60% kcal fat HFD (D12492) was presented in Supplementary Table S1. The animal experiment design plan is shown in Figure 1.

### 2.3. Histopathological and Immunohistochemical Detection

Liver and ileum tissues were obtained from each group. After being rinsed with normal saline, fresh tissues were fixed in 4% paraformaldehyde, embedded in paraffin wax, and cut into sections roughly 4 μm thick. H&E and Oil Red O staining were performed following standard protocols. Three different fields of view were observed to assess liver and intestinal pathological changes. The villus height and crypt depth were measured, and their ratio was calculated.

For immunohistochemical staining, intestinal tissues were initially exposed to specific primary antibodies overnight, and then treated with horseradish peroxidase (HRP)-labeled secondary antibodies. 3,3′-diaminobenzidine (DAB) chromogen was used to visualize HRP activity (brown-yellow positive signals). Pathological changes were photographed and recorded under a microscope.

### 2.4. Measurement of Serum Biochemical Indices

Orbital blood was collected after the experiment, and the serum was isolated via centrifugation at 3000 r/min for 15 min and then preserved at −80 °C. Concentrations of IGF-1, GH, GHRH, SST, DAO and D-LAC were measured using ELISA kits following the manufacturer’s protocols (Elabscience Biotechnology Co., Ltd., Wuhan, China).

Serum levels of ALT, AST, TG and TC were determined via colorimetric assays in accordance with the corresponding kit instructions (Nanjing Jiancheng Bioengineering Institute, Nanjing, China). The absorbance was measured using a microplate reader.

### 2.5. Assessment of Cell Proliferation

Cell proliferation of Caco-2 cells was evaluated via the CCK-8 method. Briefly, Caco-2 cells were seeded into 96-well plates and exposed to varying doses of LPS (0, 5, 10, 20, and 40 µg/mL), followed by a 24 h incubation at 37 °C. 2 h before incubation termination, cell morphology was visualized under an inverted microscope. Then, each well received 10 μL of CCK-8 reagent (1 mg/mL, Yeasen, Shanghai, China), and the plates were incubated for an additional 2 h. Finally, the absorbance values at 450 nm were determined with a microplate reader. All cell assays were performed with at least three independent biological replicates (*n* = 3), each consisting of multiple technical replicates.

### 2.6. Measurement of Transepithelial Electrical Resistance (TEER)

Caco-2 cells were plated in 6-well Transwell inserts at a density of 1 × 10^5^ cells per well. The apical compartment received 0.5 mL of complete DMEM medium (containing cells), while the basolateral compartment was filled with 1.5 mL of standard medium. The cells were maintained in an incubator at 37 °C with 5% CO_2_ for 21 d, divided into four groups: CON (DMEM medium), IGF-1 (100 ng/mL IGF-1), LPS (10 μg/mL LPS), and LPS + IGF-1 (10 μg/mL LPS + 100 ng/mL IGF-1). Medium was changed every other day for the first 7 d and daily thereafter. After forming a complete monolayer, the cells were rinsed 3 times with pre-warmed PBS. Electrodes were disinfected with 75% ethanol for 5 min and rinsed with sterile PBS before being inserted into the Transwell upper and lower chambers to measure TEER values. Three replicate measurements were performed for each sample, and the average value was derived. The final TEER value (Ω·cm^2^) was determined using the formula: (R sample − R blank) × effective membrane area.

### 2.7. Measurement of Cell Migration

Cell migration ability was assessed via the scratch assay. Briefly, logarithmic-phase Caco-2 cells were digested and plated. Upon reaching approximately 80% confluence, cells were separated into four groups, and scratches were made with a 100 μL sterile pipette tip. After washing 3 times with PBS to remove floating cells, corresponding drug solutions were added for a further 24 h incubation. Scratch status was photographed and recorded, and the scratch area, as well as the cell migration rate, were calculated using ImageJ software (version 1.54r).

### 2.8. Determination of Apoptosis

Intestinal tissue apoptosis was detected via TUNEL staining (Vazyme, Nanjing, China). Briefly, fixed intestinal tissues were treated with a permeabilizing agent, then incubated for 1 h with a reaction solution containing terminal deoxynucleotidyl transferase (TdT) and labeled dUTP. After washing, DAPI was used to counterstain the nuclei, and samples were mounted for subsequent observation under a fluorescence microscope.

For Caco-2 cell apoptosis detection, cells were plated in 96-well plates at a density of 1 × 10^5^ cells per well. When the cells reached roughly 80% confluence, they were grouped and incubated for 24 h. After being washed twice with PBS, the cells were labeled with Annexin V-FITC/PI staining solution (Vazyme, Nanjing, China) according to the manufacturer’s protocol. Apoptosis rate was assessed by detecting fluorescence signals using a flow cytometer (Becton, Dickinson, ND, USA).

### 2.9. Real-Time Quantitative PCR Analysis

Caco-2 cells were inoculated into 96-well plates at a concentration of 2 × 10^5^ cells per well. After reaching approximately 80% confluency, the cells were grouped and incubated for 24 h. After washing twice with PBS, Trizol reagent was used to isolate total RNA from both cells and tissues, followed by first-strand cDNA synthesis with a SYBR Premix Ex Taq Kit (TaKaRa, Kyoto, Japan). Real-time quantitative PCR was conducted on a rapid PCR system, with each 20 μL reaction mixture comprising specific primers and cDNA templates. GAPDH served as an endogenous reference gene, and the relative mRNA expression levels of target genes were calculated via the 2^−∆∆CT^ method. All qPCR primers are listed in Supplementary Table S2.

### 2.10. Western Blot

Total protein was measured on ileum specimens, and protein concentrations were determined with a BCA kit (Epizyme, Shanghai, China) according to the supplier’s guidelines. The extracted proteins were resolved by SDS-PAGE and electrophoretically transferred to a polyvinylidene fluoride (PVDF) membrane (Immobilon, MilliporeSigma, Burlington, MA, USA), which was then blocked with 5% BSA or skim milk for 2 h. After blocking, the membrane was incubated overnight at 4 °C with primary antibodies (ZO-1, Occludin, Claudin-1, IGF-1R, β-actin, phospho-PI3K, phospho-AKT; Immunoway, Suzhou, China), washed 4 times with TBST, and then for one hour at room temperature with an HRP-linked anti-rabbit secondary antibody. Positive bands were detected and photographed by means of an Omega LumG gel imaging system (Analytik Jena AG, Jena, Germany). The expression levels of the target proteins were analyzed quantitatively via Image J software, with β-actin as the internal control.

### 2.11. Statistical Analysis

All experimental procedures were conducted independently in triplicate. Data were processed using SPSS 19.0, while histograms were plotted with GraphPad Prism 8.0 (GraphPad, Boston, MA, USA). Results are expressed as mean ± standard error (x ± SEM). Differences between two groups were assessed using Student’s *t*-test, whereas among three or more groups were evaluated by one-way ANOVA with Tukey’s HSD method. The Pearson correlation coefficient was calculated to evaluate associations. The same letters are not significantly different, whereas different letters are significantly different; *p* < 0.05 indicates significant differences.

## 3. Results

### 3.1. HFD-Induced MASLD Rats Exhibited Intestinal Mechanical Barrier Damage

**Figure 2:**
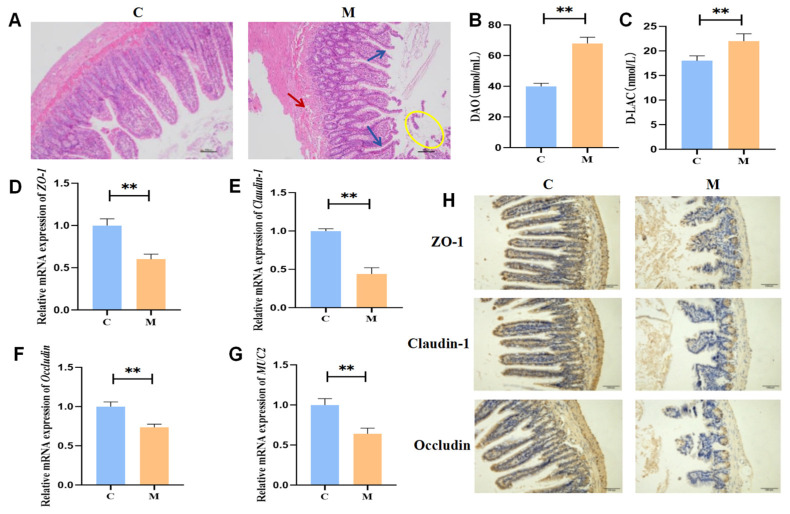
Results of intestinal mechanical barrier damage in MASLD rats. (**A**) Representative H&E staining images of ileum tissue (200×); the red arrow indicates disordered arrangement of epithelial cells, the blue arrows indicate widened intercellular spaces, and the yellow circle indicates villous fragmentation; (**B**,**C**) Serum contents of DAO and D-LAC; (**D**–**G**) mRNA expression of ZO-1, Claudin-1, Occludin and MUC2; (**H**) Representative immunohistochemical staining of ZO-1, Claudin-1 and Occludin (200×). All data are shown as means ± SEM (*n* = 6). ** *p* < 0.01.

In the present study, MASLD rat models were successfully induced via HFD feeding. H&E staining of the ileum tissue revealed well-defined mucosal layers, intact and continuous epithelium, and orderly arranged cells in the control group (C). The intestinal villi exhibited regular morphology and intact structure, with no congestion, edema or disconnection in the lamina propria and muscularis mucosae. In contrast, the model group (M) exhibited significant intestinal mucosal damage, including disordered epithelial arrangement, widened intercellular spaces, irregular villous architecture, and an uneven mucosal surface (Figure 2A). Consistent with the structural impairment, the concentrations of DAO and D-LAC were significantly higher in the M group relative to the C group (*p* < 0.01; Figure 2B,C). At the molecular level, the M group significantly reduced mRNA expression of ZO-1, Claudin-1, Occludin, and MUC2 (*p* < 0.01; Figure 2D–G). Immunohistochemical staining further verified these alterations. In the C group, ZO-1 and Claudin-1 exhibited strong and continuous distribution along the epithelial junctions, and Occludin was uniformly distributed. In the M group, the positive signals for all three proteins were markedly weakened, appearing discontinuous and fragmented (Figure 2H). In conclusion, MASLD induced by HFD demonstrated intestinal mechanical barrier damage, which provided a reliable model basis for subsequent studies on the protective effect of IGF-1.

### 3.2. The GH/IGF-1 Axis Is Dysregulated in HFD-Induced MASLD Rats

**Figure 3:**
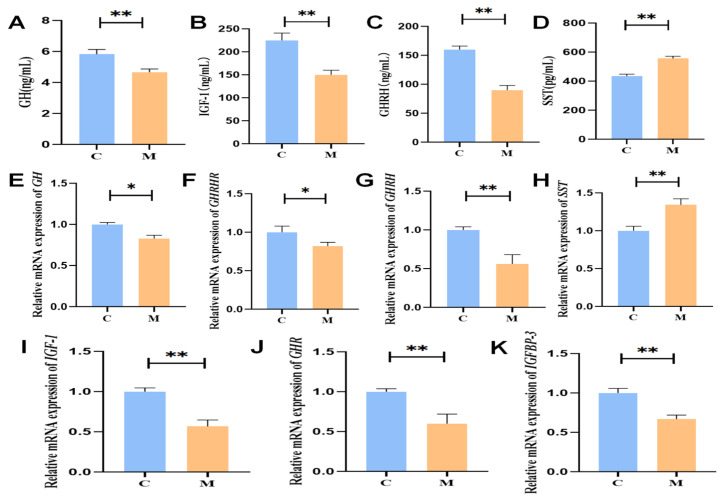
Changes in the GH/IGF-1 axis in MASLD rats. (**A**–**D**) Serum contents of GH, IGF-1, GHRH and SST; (**E**,**F**) mRNA levels of GH and GHRHR; (**G**,**H**) mRNA levels of hypothalamic GHRH and SST; (**I**) mRNA level of IGF-1; (**J**,**K**) mRNA levels of GHR and IGFBP-3. All data are expressed as means ± SEM (*n* = 6). * *p* < 0.05; ** *p* < 0.01.

To evaluate the status of the GH/IGF-1 axis in MASLD, we first measured the serum levels of its key regulatory hormones. Relative to the C group, the M group showed a marked reduction in GH, IGF-1 and GHRH levels (*p* < 0.01), along with a notable increase in SST levels (*p* < 0.01; Figure 3A–D).

To further elucidate the underlying disturbances, we analyzed the mRNA expression of relevant factors across the hypothalamic-pituitary-hepatic axis. In the pituitary, the M group exhibited significant downregulation of both GH and GHRHR mRNA (*p* < 0.05, Figure 3E,F). In the hypothalamus, the M group displayed a marked decrease in GHRH mRNA expression and a significant increase in SST mRNA levels (*p* < 0.01; Figure 3G,H), which aligned with the alterations observed in serum. In the liver, the M group showed significantly reduced mRNA expression of IGF-1, GHR, and IGFBP-3 (*p* < 0.01; Figure 3I–K). Collectively, these results demonstrated a comprehensive dysregulation of the GH/IGF-1 axis at both systemic (serum) and tissue (hypothalamus, pituitary, liver) levels in HFD-induced MASLD rats.

### 3.3. IGF-1 Was Negatively Correlated with Intestinal Barrier Damage in MASLD Rats

**Figure 4:**
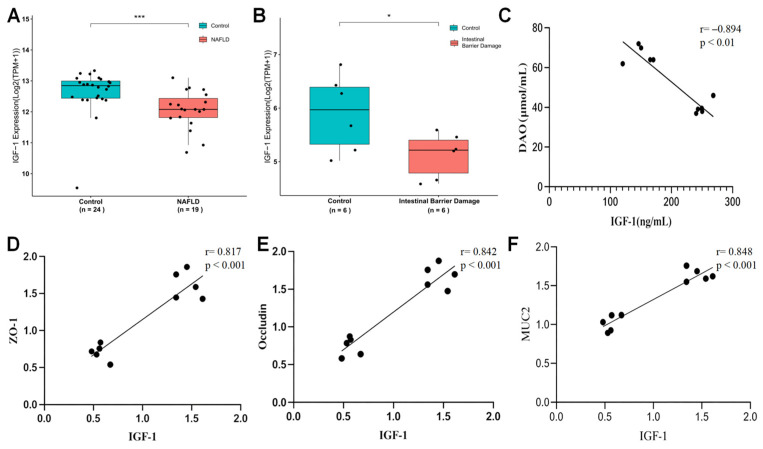
Correlation analysis of IGF-1 and intestinal barrier injury in MASLD rats. (**A**) Association between IGF-1 expression and MASLD; (**B**) Association between IGF-1 levels and intestinal barrier damage in MASLD; (**C**–**F**) Correlation between IGF-1 and DAO, ZO-1, Occludin and MUC2. * *p* < 0.05, significant difference; *** *p* < 0.001, extremely significant difference. *p* < 0.01 indicates a correlation.

To investigate the potential link between IGF-1 and intestinal barrier injury in MASLD, we first conducted a bioinformatic analysis. Differential expression analysis of the MASLD-related dataset (GSE89632) revealed a significant downregulation of IGF-1 expression in MASLD samples relative to controls (*p* < 0.01; Figure 4A). Consistently, analysis of the intestinal tissue dataset (GSE190140) showed that IGF-1 was also significantly lower in samples from subjects with MASLD-induced intestinal barrier damage (*p* < 0.01; Figure 4B).

To validate and extend this association in our experimental model, we performed Pearson correlation analysis between IGF-1 levels and key intestinal barrier indicators in HFD-induced MASLD rats. Serum IGF-1 concentrations showed a significant, strong negative correlation with serum DAO levels (*p* < 0.01; Figure 4C). Conversely, IGF-1 mRNA expression exhibited a significant and strong positive correlation with the levels of ZO-1 and Occludin, as well as MUC2 (*p* < 0.001; Figure 4D–F). These results established a clear negative correlation between IGF-1 and intestinal barrier damage in the context of MASLD, positioning IGF-1 as a potential key regulator in this pathological process.

### 3.4. IGF-1 Alleviated Liver Injury and Improved Intestinal Barrier Function in MASLD Rats

**Figure 5:**
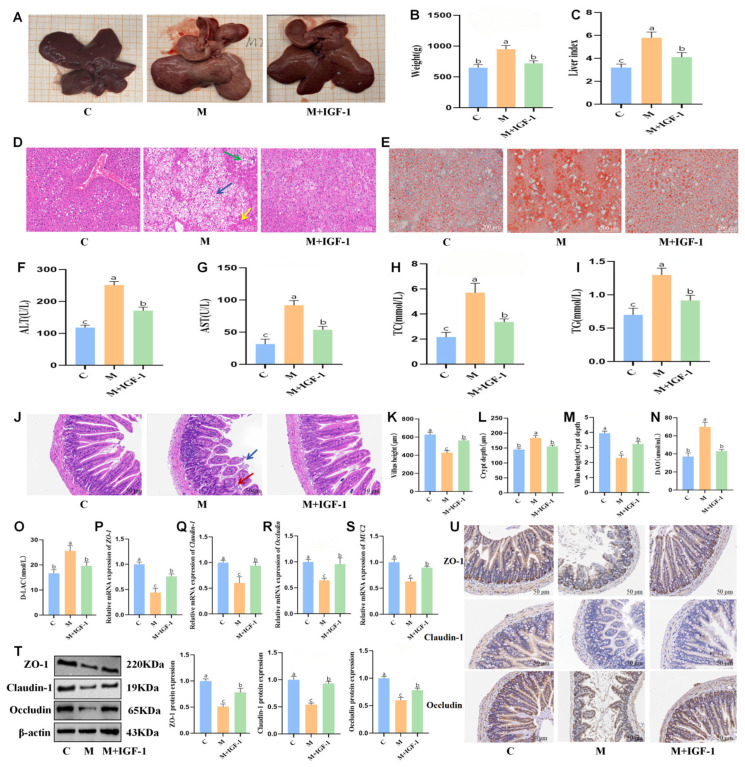
Effects of IGF-1 on liver damage and intestinal barrier function in MASLD rats. (**A**) Pathological anatomy changes in liver; (**B**) Body weight; (**C**) Liver index; (**D**) Representative images of H&E-stained liver tissue (200×); the green arrow represents hepatocyte swelling, the blue arrow represents large lipid vacuoles, and the yellow arrow represents nuclear displacement; (**E**) Representative images of oil red O-stained liver (100×); (**F**–**I**) Serum contents of ALT, AST, TC, and TG; (**J**) Representative images of H&E-stained ileum tissue (200×); the red arrow represents the disordered arrangement of the intestinal mucosa, and the blue arrow represents the shedding of the villous epithelium; (**K**–**M**) Results of villus height, crypt depth and their ratio; (**N**,**O**) Serum contents of DAO and D-LAC; (**P**–**T**) mRNA and protein expression of ZO-1, Claudin-1, Occludin, and MUC2; (**U**) Representative immunohistochemical staining images of ZO-1, Claudin-1, and Occludin (200×). All data are expressed as means ± SEM (*n* = 6). The same letters are not significantly different, whereas different letters are significantly different (*p* < 0.05).

Based on this strong correlation, we next sought to determine whether IGF-1 intervention could ameliorate intestinal barrier damage in MASLD rats. Assessment of liver morphology showed that the M group displayed hepatomegaly with a pale-yellow color, greasy surfaces, and increased hardness relative to the C group, all of which were alleviated by IGF-1 intervention (Figure 5A). Body weight and liver index were markedly elevated in the M group, whereas IGF-1 intervention reversed these trends (*p* < 0.01; Figure 5B,C). H&E and Oil Red O staining revealed severe hepatocellular steatosis, ballooning, and lipid droplet accumulation in the M group, which were significantly ameliorated by IGF-1 (Figure 5D,E). Furthermore, the serum concentrations of liver enzymes (ALT, AST) and lipids (TC, TG) were significantly elevated in the M group, and significantly decreased after IGF-1 intervention (*p* < 0.01; Figure 5F–I).

In the intestine, MASLD rats exhibited structural disorganization in the ileum, including villous atrophy, crypt deepening, and elevated serum levels of DAO and D-LAC, indicating impaired barrier function. IGF-1 intervention significantly ameliorated these morphological and functional parameters (*p* < 0.01; Figure 5J–O). At the molecular level, the expression of tight junction proteins (ZO-1, Occludin, Claudin-1) and the mucin MUC2 was generally downregulated in MASLD rats, whereas IGF-1 treatment partially restored their expression (*p* < 0.05; Figure 5P–T). Immunohistochemistry further confirmed that IGF-1 restored the continuous and intense staining pattern of tight junction proteins, which appeared weak and discontinuous in the M group (Figure 5U). In conclusion, these results suggested that IGF-1 intervention was associated with improvement in both liver injury and intestinal barrier integrity.

### 3.5. IGF-1 Promoted Proliferation and Suppressed Apoptosis of Intestinal Epithelial Cells in MASLD Rats

**Figure 6:**
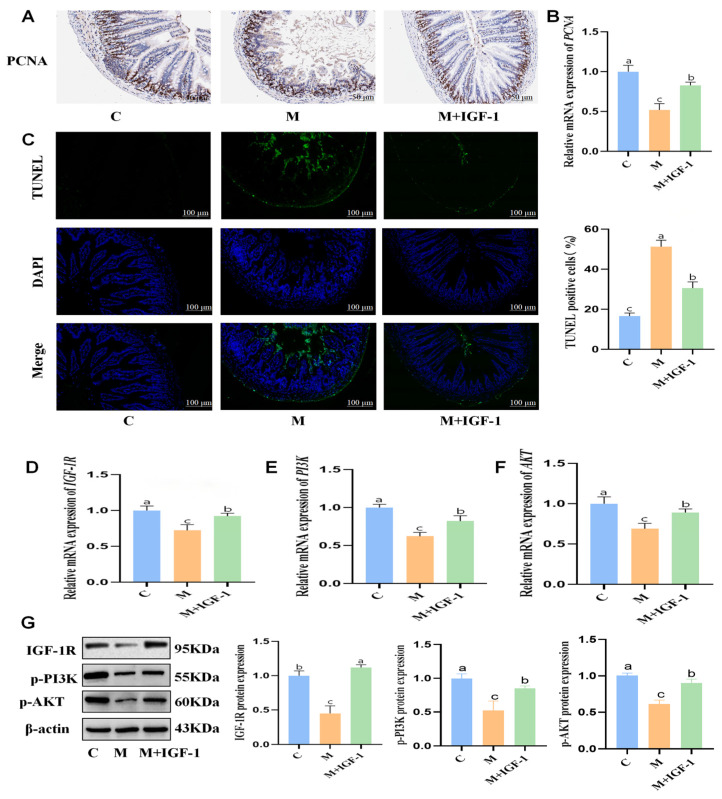
Effects of IGF-1 on intestinal epithelial cell proliferation, apoptosis and IGF-1R/PI3K/AKT signaling in MASLD rats. (**A**) Representative images of immunohistochemical staining of PCNA (200×); (**B**) mRNA level of PCNA; (**C**) TUNEL staining of cell apoptosis in ileum tissue (100×); (**D**–**F**) mRNA expression of IGF-1R, PI3K and AKT; (**G**) Protein expression levels of IGF-1R, *p*-PI3K and *p*-AKT. Data are presented as means ± SEM (*n* = 6). The same letters are not significantly different, whereas different letters are significantly different (*p* < 0.05).

To investigate the mechanisms underlying the repair of intestinal barrier integrity, the impact of IGF-1 on epithelial cell proliferation and apoptosis was assessed. Immunohistochemical staining for PCNA revealed weak and discontinuous signals in the ileum of the M group relative to the C group. In contrast, IGF-1 intervention markedly enhanced both the intensity and continuity of PCNA staining (Figure 6A). Consistent with this observation, PCNA mRNA expression was markedly downregulated in the M group (*p* < 0.01), whereas IGF-1 administration led to a significant increase (*p* < 0.05; Figure 6B).

Concurrently, TUNEL staining showed extensive apoptosis in the ileal epithelium of the M group, in contrast to the C group; IGF-1 treatment markedly diminished this apoptotic signal. Quantitative evaluation confirmed that the apoptotic rate was significantly elevated in the M group (*p* < 0.01) and was substantially decreased by IGF-1 intervention (*p* < 0.01; Figure 6C). Taken together, these findings indicated that IGF-1 restored balance between proliferation and apoptosis in intestinal epithelial cells, thereby repairing intestinal epithelial integrity.

### 3.6. IGF-1 Upregulated the Expression of IGF-1R, PI3K and AKT in the Intestinal Tissue of MASLD Rats

To detect the mechanism by which IGF-1 protects the intestinal barrier in vivo, the mRNA and phosphorylation status of IGF-1R, PI3K, and AKT in ileal tissues were detected. Relative to the C group, the M group exhibited markedly reduced mRNA and protein levels of IGF-1R, *p*-PI3K, and *p*-AKT, and IGF-1 intervention significantly reversed these changes (*p* < 0.05; Figure 6D–G). These findings suggested that IGF-1 action may be associated with alterations in IGF-1R/PI3K/AKT signaling.

### 3.7. IGF-1 Alleviated Intestinal Barrier Injury in LPS-Induced Caco-2 Cells

**Figure 7:**
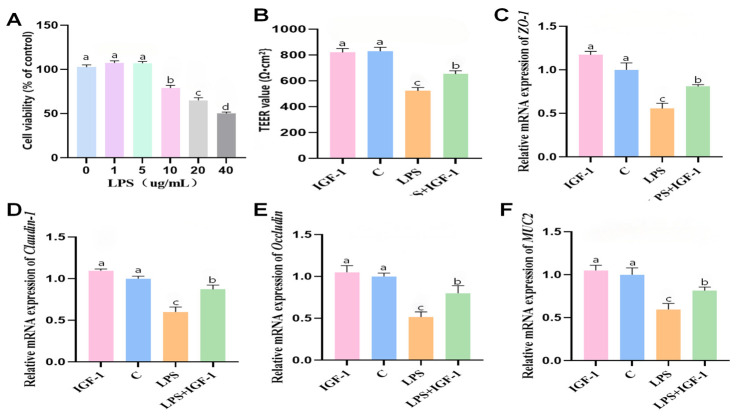
Effects of IGF-1 on intestinal barrier impairment in LPS-treated Caco-2 cells. (**A**) Cell viability; (**B**) TEER value of Caco-2 cells; (**C**–**F**) mRNA expression of ZO-1, Claudin-1, Occludin and MUC2. All data are presented as means ± SEM (*n* = 3). The same letters are not significantly different, whereas different letters are significantly different (*p* < 0.05).

To further validate the above findings, we established an in vitro intestinal barrier injury model using LPS-induced Caco-2 cells. Firstly, Caco-2 cells were exposed to gradient concentrations of LPS, and 10 μg/mL was selected as the optimal concentration for subsequent experiments based on its significant inhibitory effect (Figure 7A). Using this Caco-2 injury model established with 10 μg/mL LPS, we next evaluated the impact of IGF-1 on intestinal barrier permeability by measuring TEER. The findings revealed that LPS treatment significantly reduced TEER values, whereas IGF-1 intervention partially restored TEER (*p* < 0.05; Figure 7B). There was no notable difference in TEER values between the IGF-1 alone group and the C group.

Next, we examined whether IGF-1 improved intestinal barrier function by restoring key structural proteins of the intestinal barrier. qPCR analysis revealed that LPS treatment markedly downregulated the mRNA levels of barrier-associated genes (*p* < 0.01). However, the LPS + IGF-1 group significantly attenuated this downregulation (*p* < 0.05; Figure 7C–F). Notably, IGF-1 treatment alone did not significantly alter their expression. Collectively, these results suggested that IGF-1 was associated with the maintenance of epithelial homeostasis.

### 3.8. IGF-1 Regulated Cell Proliferation, Migration, and Apoptosis in LPS-Induced Caco-2 Cells

**Figure 8:**
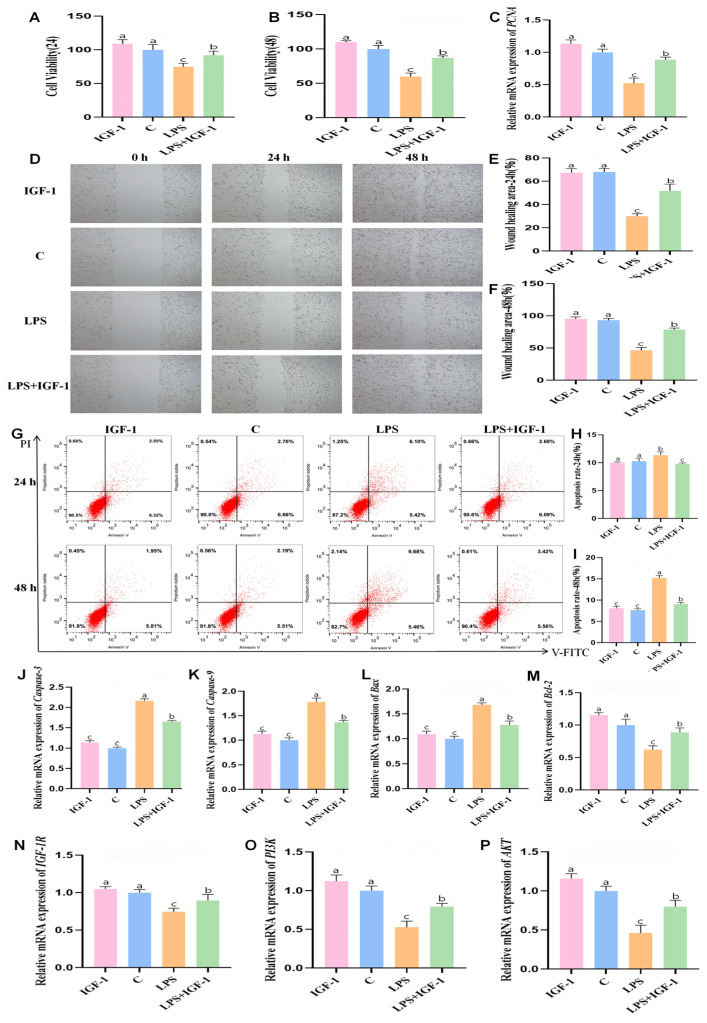
Effects of IGF-1 on cell proliferation, migration, apoptosis and IGF-1R/PI3K/AKT signaling in LPS-treated Caco-2 cells. (**A**,**B**) Proliferation rates of cells at 24 h and 48 h; (**C**) PCNA mRNA expression in cells; (**D**) Representative migration images (200×); (**E**,**F**) Migration rates at 24 and 48 h; (**G**) Representative images of cell apoptosis; (**H**,**I**) Cell apoptosis rates at 24 and 48 h; (**J**–**M**) mRNA expression of Caspase-3, Caspase-9, Bax and Bcl-2; (**N**–**P**) mRNA levels of IGF-1R, PI3K and AKT. All data are expressed as means ± SEM (*n* = 3). The same letters are not significantly different, whereas different letters are significantly different (*p* < 0.05).

The integrity of the intestinal barrier is sustained by a dynamic balance of epithelial cell proliferation, migration and apoptosis. To investigate whether IGF-1 contributes to this balance, its effects on Caco-2 cell behaviours under LPS treatment were assessed. In terms of cell proliferation, LPS treatment markedly inhibited cell growth at both 24 h and 48 h relative to the C group (*p* < 0.01). This suppression was markedly reversed by LPS + IGF-1, with a more pronounced pro-proliferative effect observed at 48 h (*p* < 0.01; Figure 8A,B). Accordingly, the mRNA expression of the proliferation marker PCNA was also drastically downregulated by LPS, and this downregulation was significantly rescued by IGF-1 (*p* < 0.01; Figure 8C), with no significant change detected in the IGF-1 alone group.

Regarding cell migration, LPS significantly inhibited cell migration at both 24 h and 48 h (*p* < 0.01; Figure 8D–F). However, the LPS + IGF-1 group markedly reversed this inhibitory effect (*p* < 0.01), while IGF-1 alone did not alter the migration rate. Collectively, these data indicated that IGF-1 restored the proliferation of LPS-induced Caco-2 cells and normalized their migration.

For cell apoptosis, LPS treatment significantly increased the cell apoptosis rate at 24 h (*p* < 0.05), and this pro-apoptotic effect was further enhanced at 48 h (*p* < 0.01). LPS + IGF-1 group markedly reduced the LPS-induced apoptosis at both time points (*p* < 0.01; Figure 8G–I). IGF-1 treatment alone did not significantly alter the apoptosis rate compared with the C group. Detection of apoptosis regulatory factors showed that LPS exposure markedly increased the mRNA expression of Caspase-3, Caspase-9, and Bax, and concurrently decreased the expression of Bcl-2 (*p* < 0.01). LPS + IGF-1 group effectively reversed these LPS-mediated alterations in gene expression (*p* < 0.01; Figure 8J–M), whereas the IGF-1 alone group exhibited no substantial differences. Collectively, these results demonstrated that IGF-1 promoted intestinal barrier repair by simultaneously stimulating proliferation, normalizing migration, and inhibiting apoptosis.

### 3.9. IGF-1 Reversed LPS-Induced Downregulation of IGF-1R, PI3K and AKT Expression

To further investigate how IGF-1 molecularly ameliorated LPS-induced intestinal barrier damage, we detected the expression of IGF-1R, PI3K, and AKT in cultured cells. As presented in Figure 8N–P, LPS exposure markedly decreased the mRNA expression levels of IGF-1R, PI3K, and AKT (*p* < 0.01). In contrast, the LPS + IGF-1 group effectively reversed this downregulation (*p* < 0.01). The result was consistent with the trend observed in vivo, which further supported the association between the action of IGF-1 and alterations in the relevant signaling pathway.

## 4. Discussion

Metabolic dysfunction-associated steatotic liver disease (MASLD) is increasingly recognized as a systemic metabolic disorder characterized by complex crosstalk between the liver and intestine through the gut–liver axis. In the present study, we identified a close association among intestinal mechanical barrier dysfunction, suppression of the GH/IGF-1 axis, and alterations in IGF-1R/PI3K/AKT signaling under MASLD conditions. Critically, exogenous IGF-1 intervention mitigated intestinal mechanical barrier function, and this restoration was associated with activation of the IGF-1R/PI3K/AKT signaling pathway. Rather than establishing a direct causal mechanism, our findings suggest that these factors may be functionally interconnected within the gut–liver axis, highlighting the importance of endocrine-barrier-signaling interactions in MASLD progression.

Consistent with previous studies, our data support the presence of intestinal barrier impairment in MASLD, characterized by structural disruption and altered barrier-related markers. Rather than reiterating individual measurements, these findings collectively indicate compromised epithelial integrity under metabolic stress. Clinical observations have reported similar abnormalities, including reduced expression of tight junction proteins and disrupted epithelial continuity in MASLD patients [19,20,21]. However, the upstream regulatory factors linking metabolic disturbance to barrier dysfunction remain incompletely understood.

Our results further indicate that suppression of the GH/IGF-1 axis is closely associated with the severity of intestinal barrier damage in MASLD. Serum GH, IGF-1 and GHRH levels were decreased, while SST was increased. At the molecular level, the mRNA expressions of GH, IGF-1, GHRH, GHRHR, GHR and IGFBP-3 were downregulated, whereas SST was upregulated. For example, hepatic inflammation in MASLD inhibited the GHR-JAK2-STAT5 pathway (a core regulator of hepatic IGF-1 synthesis), thereby reducing IGF-1 production [22]. Notably, Correlation analysis revealed that IGF-1 levels were negatively associated with markers of barrier disruption and positively associated with tight junction integrity. These findings extended the clinical observations reported by Bai et al., who found that low IGF-1 levels were associated with more severe intestinal barrier impairment [23]. Collectively, these results indicate that GH/IGF-1 axis dysfunction in MASLD is closely linked to intestinal barrier injury. This dysfunction likely arises as a consequence of hepatic metabolic disturbance, yet it may also contribute to or exacerbate the intestinal barrier pathology, reflecting the bidirectional nature of the gut–liver axis.

To further explore this relationship, we evaluated the effects of exogenous IGF-1 intervention. Our data show that IGF-1 treatment is associated with partial restoration of intestinal barrier integrity, as evidenced by improved histological structure, reduced serum DAO levels, and increased expression of tight junction proteins. In parallel, IGF-1 intervention was associated with the attenuation of hepatic steatosis and inflammation in MASLD rats. These findings are in line with the established function of IGF-1 in regulating intestinal epithelial differentiation and restoring mucosal architecture [15], and further highlight the importance of gut–liver crosstalk in MASLD [24]. While recent investigations into the gut–liver axis have productively focused on microbial and inflammatory components [25,26], the role of systemic endocrine regulators like the GH/IGF-1 axis remains less explored. For instance, Huang et al. linked microbiota to IGF-1 production but did not explore the downstream effects on intestinal barrier integrity [27]. Our findings support an emerging link between endocrine regulation and intestinal barrier integrity. Notably, IGF-1 intervention was associated with concurrent improvement in both intestinal and hepatic parameters, suggesting functional interconnection within the gut–liver axis. A plausible interpretation is that restoration of the intestinal barrier may contribute, at least in part, to the hepatic improvements by reducing the translocation of gut-derived inflammatory mediators. However, the present study design cannot disentangle the direct hepatic effects of IGF-1 from those secondary to intestinal repair, underscoring the bidirectional complexity of this axis. At the cellular level, IGF-1 intervention was associated with enhanced epithelial proliferation, reduced apoptosis, and improved barrier function, as reflected by TEER recovery in LPS-treated Caco-2 cells. These findings are consistent with previous reports demonstrating that IGF-1 supports epithelial repair processes [28]. Together, these integrated observations in which IGF-1 promotes the repair of intestinal mechanical barrier damage, a process that is correlated with broader metabolic and hepatic improvement in MASLD.

Mechanistically, our findings suggested that activation of the IGF-1R/PI3K/AKT signaling pathway was observed alongside barrier improvement in both in vivo and in vitro models. Accumulating evidence has verified that activation of the PI3K/AKT pathway enhances tight junction assembly and barrier function in multiple disease models [29]. While the PI3K/AKT pathway is well known to regulate cell survival and tight junction assembly [16], our data do not establish it as the sole or indispensable mediator of IGF-1 action. Instead, it may represent one component within a broader regulatory network linking GH/IGF-1 axis activity to intestinal barrier integrity. Future studies incorporating pathway blockade and multi-level regulatory analysis will be required to clarify this relationship.

Building upon the observed associations between IGF-1 signaling, barrier repair, and systemic improvement, it is critical to evaluate the translational relevance of this axis. From a translational perspective, it is important to consider the role of IGF-1 in the context of current MASLD therapeutic strategies. Both IGF-1 and GLP-1 signaling pathways share overlapping roles in metabolic regulation, inflammation control, and epithelial protection [30,31]. Therefore, IGF-1 may function as a complementary or downstream mediator within broader metabolic regulatory networks, rather than representing a standalone therapeutic alternative. Notably, the clinical application of IGF-1 remains limited due to safety concerns, including its mitogenic potential, highlighting the need for cautious interpretation of its translational value.

However, several limitations should be noted in the present study. First, although the IGF-1R/PI3K/AKT pathway was implicated in the observed regulatory effects, direct evidence from specific pathway inhibition assays is lacking to verify it as the critical mediator of IGF-1 action. Second, this study only evaluated the effect of a single concentration (100 ng/mL) of IGF-1 at the cellular level. Although this concentration was established as effective based on previous literature [14,32,33], and it successfully verified the activation of the target pathway and its protective effects, this design cannot clarify the dose-response relationship of IGF-1 in improving intestinal barrier function. Third, the sample size, though consistent with many preclinical studies, is relatively limited. Future studies with larger cohorts are warranted to enhance statistical power and confirm the generalizability of our findings. Future research should establish a concentration gradient of IGF-1 to determine the dose range that elicits the optimal protective effect. Future studies employing specific pathway blockade combined with multi-omics analyses will be needed to further define the core signaling cascade and regulatory networks of IGF-1. Such investigations will furnish a more solid theoretical basis for the development of precise, targeted therapies based on the GH/IGF-1 axis.

## 5. Conclusions

In conclusion, our findings indicate that IGF-1 levels are closely associated with intestinal mechanical barrier integrity in MASLD. Within the gut–liver axis, suppression of the GH/IGF-1 axis, intestinal barrier dysfunction, and alterations in IGF-1R/PI3K/AKT signaling appear to be interconnected. IGF-1 intervention was associated with improvements in barrier-related parameters, including tight junction integrity, epithelial proliferation, and apoptosis balance, accompanied by concurrent activation of IGF-1R/PI3K/AKT signaling. Overall, this study provides an integrated perspective on the potential interplay between endocrine regulation, intestinal barrier function, and intracellular signaling in MASLD. These findings may offer supportive evidence for further exploration of the GH/IGF-1 axis as a potential target in MASLD management.

## Data Availability

The raw data supporting the conclusions of this article will be made available by the authors on request.

## Supplementary Materials

The following supporting information can be downloaded at: https://www.mdpi.com/article/10.3390/nu18111667/s1, Table S1: Ingredient composition of the 60% kcal HFD; Table S2: Primer sequences used for qRT-PCR.
