# Supplementary material for: Insulin-like Growth Factor 1 Ameliorates Intestinal Barrier Dysfunction in MASLD via IGF-1R/PI3K/AKT Signaling
Source: Nutrients. 2026 May 22;18(11):1667. doi: 10.3390/nu18111667 (PMC13257688; doi:10.3390/nu18111667)
Supplement: Supplementary file 1 [file nutrients-18-01667-s001.zip › nutrients-4292814-supplementary.pdf]

Supplementary Tables

Table S1 Ingredient composition of the 60% kcal HFD

Ingredient	Gm%	Kcal%
Casein	200	800
L-Cystine	3	12
Corn Starch	0	0
Maltodextrin 10	125	500
Sucrose	68.8	275.2
Cellulose	50	0
Soybean Oil	25	225
Lard	245	2205
Mineral Mix S10026	10	0
Dicalcium Phosphate	13	0
Calcium Carbonate	5.5	0
Potassium Citrate	16.5	0
Vitamin Mix V10001	10	40
Choline Bitartrate	2	0
FD&C Yellow Dye #5	0	0
FD&C Red Dye #40	0	0
FD&C Blue Dye #1	0.05	0
**Total**	**773.85**	**4057**

Table S2 Primer sequences used for qRT-PCR

Source	Gene	Forward (5'-3')	Reverse (3'-5')
Rat	*Zo-1*	AACTGGGCTCTTGGCTTGCTATTC	TCCAGAAGTCAGCACGGTCTCC
	Occludin	AACTTCGCCTGTGGATGACTTCAG	GACCTTCCTGCTCTTCCCTTTGC
	Claudin-1	CTTCTGGGTTTCATCCTGGCTTCG	CCTGAGCAGTCACGATGTTGTCC
	MUC2	CTTGCTGAACTGGAGGCTGATGG	CCGTTTTCTTATGGGCTGGCTCTC
	GH	GCTTCTCGCTGCTGCTCATCC	ATGCCCTCTTCCAGGTCCTTCAG
	GHRHR	TGCCACCATGACCAACTTCAGC	CGAGAACCAGCCACCAGAAAGC
	SST	CTGGCTTTGGGCGGTGTCAC	GTTGGGCTCGGACAGCAGTTC
	GHRH	CTCTGGGTGTTCTTTGTGCTCCTC	CTGCTGGTGAAGATGGCGTCTG
	IGFBP3	AGAAACAGTGTCGCCCTTCCAAAG	AGGCAATGCACGTCGTCTTTCC
	GHR	ACATGCTGCCAGTGTGTCCATC	ACATGCTGCCAGTGTGTCCATC
	IGF-1	TGAGGAGGCTGGAGATGTACTGTG	CGATAGGGGCTGGGACTTCTGAG
	GAPDH	GCCATCACAGCCACACAGAAGA	CGGCAGGTCAGGTCAACAACAG
Human	*ZO-1*	AACTGGGCTCTTGGCTTGCTATTC	TCCAGAAGTCAGCACGGTCTCC
	Occludin	AACTTCGCCTGTGGATGACTTCAG	GACCTTCCTGCTCTTCCCTTTGC
	Claudin-1	CTTCTGGGTTTCATCCTGGCTTCG	CCTGAGCAGTCACGATGTTGTCC
	IGF-1R	TGCTGACCTCTGTTACCTCTCCAC	GTCTTCTCACACATCGGCTTCTCC
	Bcl-2	TGGGATGCCTTTGTGGAACTG	TTCAGAGACAGCCAGGAGAAATC
	Bax	CAGGATGCGTCCACCAAGAAG	TCCATGTTACTGTCCAGTTCGTC
	Caspase-9	TTGGTGATGTCGGTGCTCTTG	CACGGCAGAAGTTCACATTGTTG
	Caspase-3	TGAGACAGACAGTGGTGTTGATG	ATCCTTTGAATTTCGCCAAGAATAAT AAC
	PI3K	GAAGCACCTGAATAGGCAAGTCG	GAGCATCCATGAAATCTGGTCGC
	GAPDH	AACTTTGGTATCGTGGAAGGACTC	CAGTAGAGGCAGGGATGATGTTC
	MUC2	AACCACCTCACCAACCTCCTCAG	TCCAGAATCCAGCCAGCCAGTC
	PCNA	GAAGGTGTTGGAGGCACTCAAGG	GCAGCGGTAGGTGTCGAAGC
